# How effective are the components of active management of the third stage of labor?

**DOI:** 10.1186/1471-2393-13-46

**Published:** 2013-02-21

**Authors:** Wendy R Sheldon, Jill Durocher, Beverly Winikoff, Jennifer Blum, James Trussell

**Affiliations:** 1 Office of Population Research, Princeton University, Princeton, NJ, USA; 2 Gynuity Health Projects, 15 East 26th Street, Suite 801, New York, NY, USA; 3 The Hull York Medical School, Hull, England

**Keywords:** Post-partum hemorrhage, Oxytocin, Controlled cord traction, Uterine massage, Active management

## Abstract

**Background:**

Active management of the third stage of labor is recommended for the prevention of post-partum hemorrhage and commonly entails prophylactic administration of a uterotonic agent, controlled cord traction, and uterine massage. While oxytocin is the first-choice uterotonic, it is not known whether its effectiveness varies by route of administration. There is also insufficient evidence regarding the value of controlled cord traction or uterine massage. This analysis assessed the independent and combined effectiveness of all three interventions, and the effect of route of oxytocin administration on post-partum blood loss.

**Methods:**

Secondary data were analyzed from 39202 hospital-based births in four countries and two clinical regimens: one in which oxytocin was administered following delivery of the baby; the other in which it was not. We used logistic regression to examine associations between clinical and demographic variables and post-partum blood loss ≥ 700 mL.

**Results:**

Among those with no oxytocin prophylaxis, provision of controlled cord traction reduced hemorrhage risk by nearly 50% as compared with expectant management (P < 0.001). Among those with oxytocin prophylaxis, provision of controlled cord traction reduced hemorrhage risk by 66% when oxytocin was intramuscular (P < 0.001), but conferred no benefit when oxytocin was intravenous. Route of administration was important when oxytocin was the only intervention provided: intravenous administration reduced hemorrhage risk by 76% as compared with intramuscular administration (P < 0.001); when combined with other interventions, route of administration had no effect. In both clinical regimens, uterine massage was associated with increased hemorrhage risk.

**Conclusions:**

Recommendations for active management of the third stage of labor should account for setting-related differences such as the availability of oxytocin and its route of administration. The optimal combination of interventions will vary accordingly.

## Background

Post-partum hemorrhage is a leading cause of maternal death in low resource settings [1]. Clinical guidelines for the prevention of post-partum hemorrhage widely recommend provision of a package of interventions known collectively as Active Management of the Third Stage of Labor (AMTSL) [2-4]. Although there is some variation across AMSTL guidelines, the interventions commonly include prophylactic administration of a uterotonic agent, controlled traction of the umbilical cord, and uterine massage. AMTSL is intended to reduce post-partum blood loss through expediting placental delivery and preventing uterine atony [5].

Uterotonic agents are administered shortly following delivery of the baby to stimulate uterine contractions. Oxytocin is the first-choice agent due to its high efficacy and low incidence of adverse side effects [6-9]. In a 2001 systematic review of seven randomized or quasi-randomized controlled trials involving more than 3000 women, oxytocin prophylaxis was associated with a 50% reduction in the risk of post-partum bleeding ≥ 500 mL, as compared with no uterotonics [6]. Oxytocin can be administered intravenously or intramuscularly and it has been widely assumed that its effect on post-partum blood loss does not vary with route of administration [7]. This assumption is based largely on a 1972 pharmacokinetic study (N = 26) that found little difference in the absorption rates of intramuscular (IM) and intravenous (IV) oxytocin [10]. Since then, no published studies have further examined this question [11].

Controlled cord traction is intended to facilitate placental separation and delivery. It involves manual application of gentle downward tension on the umbilical cord while maintaining counter pressure on the uterus, and is administered in conjunction with uterine contractions [2,3,12]. So far no studies have assessed the independent effect of controlled cord traction on post-partum blood loss, and only a few have assessed its effect in combination with other AMTSL components. Most recently, a large randomized controlled trial involving more than 24000 participants found that provision of controlled cord traction had only a small, non-significant effect on the risk of severe hemorrhage among women who were already receiving oxytocin prophylaxis (intravenous or intramuscular) [13]. In contrast, a 1997 trial involving more than 1600 women found that controlled cord traction combined with intramuscular oxytocin reduced the risk of post-partum bleeding ≥ 500 mL by 50% as compared with expectant management [14]. Lastly a 2009 pilot study (N = 204) found a non-significant decrease in median blood loss among those who received all three AMTSL components, as compared with receipt of uterine massage plus oxytocin (intramuscular or intravenous) [15].

Uterine massage is believed to stimulate uterine contractions through prompting the release of prostaglandins. As a result, in 2004 the International Confederation of Midwives (ICM) and International Federation of Gynaecologists and Obstetricians (FIGO) added uterine massage to their updated AMTSL guidelines [2]. To date, two trials have assessed the effect of uterine massage on post-partum blood loss. The first was a pilot study (N = 200) in which provision of all three AMTSL interventions was associated with a non-significant decrease in the risk of post-partum hemorrhage (> 500 mL), as compared with oxytocin plus controlled cord traction [16]. In the second trial uterine massage was associated with increased blood loss, although treatment was initiated before placental delivery rather than after, as per current AMTSL guidelines [17].

Numerous studies have also attempted to assess the cumulative effectiveness of the full AMTSL package. Findings from a 2010 systematic review of three randomized controlled trials, all comparing active with expectant management, indicated that provision of all three interventions reduced the average risk of severe post-partum hemorrhage (≥ 1000 mL) by two-thirds [5]. The studies in this review, however, used an early definition of AMTSL that did not include uterine massage.

There are a number of important, unanswered questions about the effect of the AMTSL components on post-partum blood loss. While this is partly due to a paucity of evidence, it also results from heterogeneity across studies in selection of treatment and control groups, measurement of blood loss (e.g. visual assessment versus more objective means), and variation over time in the AMTSL package. The primary objectives of this analysis, therefore, were to assess systematically the independent and combined effectiveness of all three current AMTSL components, and to examine the effect of route of oxytocin administration on measured post-partum blood loss.

## Methods

Data were collected as part of two trials that were conducted simultaneously and compared two uterotonic options (oxytocin versus misoprostol) for the treatment of post-partum hemorrhage, the details of which have been reported elsewhere [18,19]. The trials took place between August, 2005 and January, 2008 and involved nine secondary- and tertiary-level hospitals from five countries and two distinct clinical regimens. In the first regimen oxytocin was not routinely provided during the third stage of labor, while in the second oxytocin was routinely provided. Study sites included hospitals in Egypt (one), Ecuador (one) and Vietnam (two) in the no oxytocin prophylaxis regimen; and Burkina Faso (one), Egypt (one), Turkey (one) and Vietnam (two) in the oxytocin prophylaxis regimen. The original research was approved by the Western Institutional Review Board (Seattle, WA, USA) and relevant institutional review boards in participating countries.

All women were screened for eligibility and provided written informed consent upon admission to the hospital labor ward. Women were excluded if they delivered by caesarean section, delivery occurred outside the hospital facility, or they were enrolled in the no oxytocin prophylaxis regimen but received uterotonics for any reason during labor. The study protocols were identical with exception of the provision of oxytocin prophylaxis. For women in the oxytocin prophylaxis regimen, oxytocin (10 IU or 5 IU) was administered intramuscularly or intravenously in accordance with standard hospital practice. For women in both clinical regimens, controlled cord traction and uterine massage were provided at the discretion of each site and in accordance with standard hospital practices.

Information about basic demographic and obstetric characteristics, pre-partum hemoglobin concentrations, post-partum blood loss, provision of all AMTSL components, route of oxytocin administration (if applicable), and duration of the third stage of labor were recorded for each participant. Hemoglobin was measured using a Hemocue device (Hemocue®, Ängelholm, Sweden). Blood loss was measured using a polyurethane receptacle with calibrated funnel (Brasss-V Drapes®, Excellent Fixable Drapes, India) beginning immediately after birth of the baby and continuing for the first hour post-partum or, if hemorrhage was diagnosed, until the cessation of active bleeding. Treatment for hemorrhage was provided if bleeding reached 700 mL within the first hour post-partum or, prior to that, by clinical judgment.

For this analysis we used data from eight of the nine study sites. We excluded all 1625 participants from Quito, Ecuador, the only high elevation site in the study, due to atypical blood loss patterns across the AMTSL groups studied (overall, rates of hemorrhage were 30% in Quito versus 2–12% at all other sites). We examined bivariate relationships between clinical and demographic variables and outcomes of mean and median post-partum blood loss, blood loss ≥ 500 mL, and blood loss ≥ 700 mL. For the multivariate analyses we used logistic regression with an outcome of blood loss ≥ 700 mL in order to capture the more serious cases, and because most women diagnosed with post-partum hemorrhage received treatment at this level of blood loss. We also conducted multivariate analyses using an outcome of blood loss ≥ 500 mL, and the results (not shown) were largely consistent with those obtained using the 700 mL cutpoint.

All analyses were stratified by receipt of oxytocin prophylaxis in order to adjust for heterogeneity that may have been due to clinical regimen. We were unable to include site controls directly in the regression models due to lack of within-site variation in the provision of AMTSL components.

The protocol for this analysis was approved by Princeton University’s Institutional Review Board on 19 December 2009, protocol number 0000004672.

## Results

8427 women were enrolled in the no oxytocin prophylaxis regimen, 8221 of whom were included in these analyses. Among the 206 excluded from analysis, 45 had caesarean sections, 153 were missing information on post-partum blood loss, and eight were missing information on AMTSL components. 31180 women were enrolled in the oxytocin prophylaxis regimen, 30981 of whom were included in these analyses. Among the 199 excluded from analysis, four delivered outside the hospital, 79 had caesarean sections, 45 were missing information on post-partum blood loss, 39 were missing information on AMTSL components, and nine did not receive oxytocin during the third stage of labor. 23 others received oxytocin plus uterine massage and were excluded due to the small number of participants for that AMTSL group.

The demographic and obstetric characteristics of study participants by AMTSL group are summarized in Table 1. There were important country-level differences in provision of AMTSL components that were largely site-driven. There were few other demographic differences across AMTSL groups with exception of educational attainment, which primarily reflected country-level disparities; and obstetric practices such as the timing of umbilical cord clamping and receipt of epidural and labor induction/augmentation, all of which reflected site-level differences.

**Table 1:** Demographic and obstetric characteristics of study participants by receipt of AMTSL components (N = 39202)

	**No oxytocin prophylaxis (N = 8221)**				**Oxytocin prophylaxis (N = 30981)**		
	**No AMTSL components (n = 1832)**	**UM only (n = 959)**	**CCT only (n = 4014)**	**CCT + UM (n = 1416)**	**Oxytocin only (n = 3638)**	**Oxytocin + CCT (n = 6898)**	**Oxytocin + CCT + UM (n = 20445)**
**Country of residence**							
Burkina Faso	0 (0.0)	0 (0.0)	0 (0.0)	0 (0.0)	0 (0.0)	9 (0.1)	3684 (18.0)
Egypt	2 (0.1)	2 (0.2)	3979 (99.1)	1385 (97.8)	0 (0.0)	0 (0.0)	16305 (79.8)
Turkey	0 (0.0)	0 (0.0)	0 (0.0)	0 (0.0)	2385 (65.6)	544 (7.9)	11 (0.1)
Vietnam	1830 (99.9)	957 (99.8)	35 (0.9)	31 (2.2)	1253 (34.4)	6345 (92.0)	445 (2.2)
**Mean age in years ± SD**	26.2 ± 4.9	26.2 ± 4.8	26.1 ± 5.1	26.9 ± 4.8	25.6 ± 5.2	25.6 ± 5.0	25.3 ± 5.2
**Educational attainment**							
No education	60 (3.3)	12 (1.3)	1122 (28.0)	357 (25.2)	102 (2.8)	161 (2.3)	8190 (40.1)
Primary	660 (36.1)	348 (36.4)	1178 (29.4)	439 (31.0)	1877 (51.7)	3384 (49.2)	2763 (13.5)
Secondary or higher	1111 (60.7)	597 (62.4)	1711 (42.7)	620 (43.8)	1655 (45.5)	3338 (48.5)	9477 (46.4)
**Currently married**	1812 (99.0)	952 (99.3)	4009 (99.9)	1415 (100.0)	3626 (99.7)	6830 (99.3)	20258 (99.1)
**Parity**							
0	930 (50.9)	489 (51.0)	1706 (42.5)	543 (38.4)	1759 (48.4)	3712 (53.8)	9175 (44.9)
1-3	895 (49.0)	468 (48.9)	2133 (53.1)	795 (56.2)	1860 (51.2)	3163 (45.9)	10084 (49.4)
4+	1 (0.1)	1 (0.1)	175 (4.4)	77 (5.4)	17 (0.5)	21 (0.3)	1159 (5.7)
**Multiple pregnancy**	26 (1.4)	9 (0.9)	31 (0.8)	26 (1.8)	39 (1.1)	61 (0.9)	351 (1.7)
**Mean gestational age at delivery in weeks ± SD**	38.7 ± 1.6	39.0 ± 1.2	39.5 ± 1.4	39.5 ± 1.3	39.0 ± 1.6	39.4 ± 1.3	39.1 ± 1.8
**Prior known post-partum hemorrhage**	15 (0.8)	4 (0.4)	4 (0.1)	3 (0.2)	56 (1.5)	39 (0.6)	165 (0.8)
**Mean pre-delivery hemoglobin in g/dL ± SD**	11.9 ± 1.4	12.0 ± 1.1	11.1 ± 1.2	10.9 ± 1.4	12.1 ± 1.5	12.3 ± 1.3	11.0 ± 1.1
**Early cord clamping performed**	15 (0.8)	101 (10.5)	3981 (99.2)	1396 (98.6)	2594 (71.3)	6841 (99.2)	20428 (99.9)
**Epidural given**	37 (2.0)	69 (7.2)	2 (0.1)	1 (0.1)	24 (0.7)	42 (0.6)	21 (0.1)
**Labor induced or augmented**	0 (0.0)	0 (0.0)	0 (0.0)	0 (0.0)	1968 (54.3)	681 (9.9)	11898 (58.3)

Data are n (%) unless otherwise indicated. AMTSL denotes active management of the third stage of labor; *CCT*, controlled cord traction; *UM*, uterine massage; and *SD*, standard deviation.

The distribution of post-partum blood loss across AMTSL groups is summarized in Table 2. The variation in median blood loss was greatest in the no oxytocin prophylaxis regimen and ranged from 300 mL among those receiving uterine massage only, to 135 mL among those receiving controlled cord traction only. Within the oxytocin prophylaxis regimen, median blood loss was highest (300 mL) among those who received intramuscular oxytocin only or intramuscular oxytocin plus controlled cord traction, and lowest (200 mL) among those who received all three AMTSL components, irrespective of route of oxytocin administration.

**Table 2:** Distribution of post-partum blood loss (mL) by receipt of AMTSL components (N = 39184)*

	**n**	**Mean total blood loss (SD)**	**Median total blood loss (IQR)**	**Range**
**No oxytocin prophylaxis**				
No AMTSL components	1832	325 (240)	250 (200)	50-1800
UM only	959	368 (268)	300 (250)	50-2000
CCT only	4014	181 (167)	135 (95)	10-1550
CCT + UM	1416	247 (255)	145 (125)	25-1350
**Oxytocin prophylaxis**				
*Intramuscular administration*				
Oxytocin only	2853	335 (234)	300 (200)	25-3000
Oxytocin + CCT	6103	279 (115)	300 (100)	100-2500
Oxytocin + CCT + UM	4085	240 (195)	200 (180)	0-2250
*Intravenous administration*				
Oxytocin only	785	277 (175)	250 (200)	50-2000
Oxytocin + CCT	794	272 (149)	250 (120)	50-1700
Oxytocin + CCT + UM	16343	235 (152)	200 (100)	30-2000

*Total blood loss was recorded upon cessation of active bleeding.

AMTSL denotes active management of the third stage of labor; *CCT*, controlled cord traction; *UM*, uterine massage; *SD*, standard deviation; and *IQR*, inter-quartile range.

The incidence of post-partum hemorrhage across AMTSL groups is presented in Table 3. With no oxytocin prophylaxis, the rate of blood loss ≥ 700 mL was highest among those who received uterine massage plus controlled cord traction (13.8%) and lowest among those who received controlled cord traction only (4.9%). With oxytocin prophylaxis, the rate of blood loss ≥ 700 mL was highest among those who received oxytocin only by intramuscular administration (3.8%) and lowest among those who received oxytocin only by intravenous administration (1.0%).

**Table 3:** Incidence of post-partum hemorrhage by severity of blood loss and receipt of AMTSL components (N = 39184)

	**n**	**Blood loss ≥ 500 mL**	**Blood loss ≥ 700 mL**
**No oxytocin prophylaxis**		**n = 932**	**n = 638**
No AMTSL components	1832	302 (16.5)	154 (8.4)
UM only	959	219 (22.8)	93 (9.7)
CCT only	4014	204 (5.1)	196 (4.9)
UM + CCT	1416	207 (14.6)	195 (13.8)
**Oxytocin prophylaxis**		**n = 1740**	**n = 807**
*Intramuscular administration*			
Oxytocin only	2853	560 (19.6)	109 (3.8)
Oxytocin + CCT	6103	147 (2.4)	90 (1.5)
Oxytocin + CCT + UM	4085	223 (5.5)	110 (2.7)
*Intravenous administration*			
Oxytocin only	785	99 (12.6)	8 (1.0)
Oxytocin + CCT	794	58 (7.3)	9 (1.1)
Oxytocin + CCT + UM	16343	653 (4.0)	481 (2.9)

Data are n (%). AMTSL denotes active management of the third stage of labor; *CCT*, controlled cord traction; *UM*, uterine massage; and *PPH*, post-partum hemorrhage.

Table 4 shows logistic regression results for both clinical regimens using an outcome of blood loss ≥ 700 mL. In the no oxytocin prophylaxis regimen, provision of controlled cord traction reduced post-partum hemorrhage risk by nearly 50% as compared with no AMTSL components (OR = 0.53, 95% CI 0.42–0.66). Provision of uterine massage was associated with increased hemorrhage risk, although these differences were only statistically significant for those receiving controlled cord traction plus massage (OR = 1.66, 95% CI 1.31–2.10). Other predictors of post-partum hemorrhage risk were nulliparity (OR = 1.24, 95% CI 1.04–1.48) and anemia (OR = 1.19, 95% CI 1.00–1.42).

**Table 4:** Factors associated with post-partum hemorrhage: Results from logistic regression analyses

	**Blood loss ≥ 700 mL**			
	**No oxytocin prophylaxis (N = 8154)**		**Oxytocin prophylaxis (N = 30569)**	
	**OR (95% CI)**	**p-value**	**OR (95% CI)**	**p-value**
Receipt of AMTSL components*				
None	ref	-	-	-
UM only	1.14 (0.87-1.50)	0.349	-	-
CCT only	0.53 (0.42-0.66)	0.000	-	-
CCT + UM	1.66 (1.31-2.10)	0.000	-	-
Oxytocin (IM) only	-	-	ref	-
Oxytocin (IM) + CCT	-	-	0.33 (0.25-0.45)	0.000
Oxytocin (IM) + CCT + UM	-	-	0.65 (0.48-0.88)	0.006
Oxytocin (IV) only	-	-	0.24 (0.12-0.50)	0.000
Oxytocin (IV) + CCT	-	-	0.28 (0.14-0.55)	0.000
Oxytocin (IV) + CCT + UM	-	-	0.79 (0.62-0.99)	0.044
**Labor induced/augmented**	-	-	0.91 (0.76-1.09)	0.284
**Parity**				
0	1.24 (1.04-1.48)	0.016	2.36 (2.01-2.76)	0.000
1-3	ref	-	ref	-
4+	0.61 (0.32-1.16)	0.130	0.51 (0.29-0.88)	0.017
**Multiple pregnancy**	1.33 (0.68-2.62)	0.401	1.62 (0.97-2.70)	0.065
**Gestational age at delivery weeks**				
<37	0.40 (0.22-0.73)	0.002	0.32 (0.20-0.51)	0.000
37-41	ref	-	ref	-
42+	0.82 (0.46-1.45)	0.495	1.48 (0.93-2.36)	0.102
Anemia**	1.19 (1.00-1.42)	0.049	1.28 (1.11-1.49)	0.001
**Prior known post-partum hemorrhage**	2.18 (0.74-6.48)	0.159	5.17 (3.21-8.31)	0.000
**Age (years)**				
<20	1.24 (0.89-1.73)	0.195	0.78 (0.61-0.99)	0.041
20-34	ref	-	ref	-
35+	0.94 (0.66-1.32)	0.715	1.46 (1.07-1.99)	0.017
**Educational attainment**				
No education	ref	-	ref	-
Primary school	0.87 (0.67-1.13)	0.288	1.14 (0.91-1.43)	0.250
Secondary or higher	1.03 (0.81-1.32)	0.786	1.20 (1.00-1.44)	0.045

AMTSL denotes active management of the third stage of labor; *CCT*, controlled cord traction; and *UM*, uterine massage, IM, intramuscular; and IV, intravenous.

*Additional tests for differences between AMTSL groups in the oxytocin prophylaxis regimen were obtained by re-running the regression model with alternate reference categories. Results were as follows:

a. Oxy (IV) + CCT vs. oxy (IV) (ref): OR = 1.13, 95% CI 0.43-2.96.

b. Oxy (IM) + CCT + UM vs. oxy (IM) + CCT (ref): OR = 1.94, 95% CI 1.43-2.65.

c. Oxy (IV) + CCT + UM vs. oxy (IV) + CCT (ref): OR = 2.86, 95% CI 1.46-5.59.

d. Oxy (IV) + CCT vs. oxy (IM) + CCT (ref): OR = 1.21, 95% CI 0.60-2.46.

e. Oxy (IM) + CCT + UM vs. oxy (IV) + CCT + UM (ref): OR = 0.83, 95% CI 0.64-1.07.

**Anemia was defined as pre-delivery hemoglobin of < 11.0 g/dL.

In the oxytocin prophylaxis regimen, provision of controlled cord traction reduced post-partum hemorrhage risk by 66% when oxytocin was administered intramuscularly (OR = 0.33, 95% CI 0.25–0.45), but conferred no benefit when oxytocin was administered intravenously (OR = 1.13, 95% CI 0.43-2.96)^a^. Uterine massage, on the other hand, was consistently associated with increased post-partum hemorrhage risk. When comparing those who received all three AMTSL components with those who received oxytocin plus controlled cord traction, the risk of blood loss ≥ 700 mL was nearly twice as high among those with intramuscular oxytocin (OR 1.94, 95% CI 1.43–2.65)^*^, and nearly three times as high among those with intravenous oxytocin (OR = 2.86, 95% CI 1.46–5.59)^*^.

Route of oxytocin administration was important when oxytocin was the only intervention provided: in such cases intravenous administration reduced hemorrhage risk by 76% as compared with intramuscular administration (OR = 0.24, 95% CI 0.12-0.50). Route of administration had no effect, however, when oxytocin was combined with other active management interventions. There were no differences in the relative risks of blood loss ≥ 700 mL among those who received oxytocin (IV) plus controlled cord traction, as compared with oxytocin (IM) plus controlled cord traction (OR = 1.21, 95% CI 0.60-2.46)^*^; or for receipt of oxytocin (IM) plus controlled cord traction and uterine massage, as compared with oxytocin (IV) plus controlled cord traction and uterine massage (OR = 0.83, 95% CI 0.64-1.07)^*^.

Other factors associated with post-partum blood loss ≥ 700 mL in the oxytocin prophylaxis regimen were nulliparity (OR = 2.36, 95% CI 2.01–2.76), anemia (OR = 1.28, 95% CI 1.11–1.49), prior post-partum hemorrhage (OR = 5.17, 95% CI 3.21–8.31), age 35 or older (OR = 1.46, 95% CI 1.07–1.99), and completion of secondary school or higher (OR = 1.20, 95% CI 1.00–1.44). In contrast with prior research, labor induction/augmentation did not increase bleeding risk and, in both clinical regimens, neither did multiple pregnancy [20-22]. Early clamping of the umbilical cord was also not significantly associated with hemorrhage risk in either clinical regimen and was thus dropped from the final regression models. These results were consistent with those from prior research [23].

Table 5 shows the predicted probabilities of post-partum bleeding ≥ 700 mL for all AMTSL groups. In general, the predicted probability of blood loss ≥ 700 mL was considerably higher among all groups in the no oxytocin regimen, as compared with those in the oxytocin prophylaxis regimen. There was also greater variability in predicted hemorrhage risk among those who did not receive oxytocin prophylaxis, with probabilities ranging from 4.8% for receipt of controlled cord traction, to 13.7% for receipt of uterine massage plus controlled cord traction. In the oxytocin prophylaxis regimen, the predicted probability of hemorrhage ranged from 1.0% for receipt of oxytocin (IV) only to 3.9% for receipt of oxytocin (IM) only.

**Table 5:** Predicted probability of post-partum blood loss ≥ 700 mL by AMTSL components and route of oxytocin administration

	**Predicted probability**
**No oxytocin prophylaxis**	
No AMTSL components	8.8%
UM only	9.9%
CCT only	4.8%
UM + CCT	13.7%
**Oxytocin prophylaxis**	
*Intramuscular administration*	
Oxytocin only	3.9%
Oxytocin + CCT	1.3%
Oxytocin + CCT + UM	2.6%
*Intravenous administration*	
Oxytocin only	1.0%
Oxytocin + CCT	1.1%
Oxytocin + CCT + UM	3.1%

Predictions were generated from regression models summarized in Table 4. They reflect the average probability of post-partum blood loss ≥700mL, assuming that all study participants in each clinical regimen received each AMTSL treatment but otherwise retained their observed characteristics.

## Discussion

This is the first systematic assessment of the relative effectiveness of almost every possible combination of AMTSL interventions. There were important findings related to the provision of controlled cord traction. Our results suggest this intervention plays an important role in preventing post-partum hemorrhage under two circumstances: 1) when oxytocin prophylaxis is unavailable during the third stage of labor, and 2) when oxytocin prophylaxis is available but administered intramuscularly. On the other hand, it may be unnecessary if oxytocin prophylaxis is administered intravenously. Our findings are especially relevant in light of recent evidence that suggests controlled cord traction has little effect on post-partum blood loss when combined with oxytocin [13]. In this study, however, oxytocin was provided intramuscularly and intravenously, and the analyses did not the potential effect of route of oxytocin administration. Our findings are also important in light of recent concerns about whether controlled cord traction increases risks for uterine inversion or placental separation from the umbilical cord [15]. There were no reported complications related to AMTSL practices in either clinical regimen. However, the providers administering controlled cord traction were all highly skilled physicians and nurses working in hospital settings. On the other hand, this procedure may be most valuable in non-hospital settings where oxytocin is either unavailable or available by IM injection only. Since providers in these settings often have less training than those in hospital settings, this could increase procedure-related complication risks.

We found no evidence to support provision of uterine massage for the prevention of post-partum hemorrhage. In fact, our findings uniformly suggest that uterine massage either increases post-partum blood loss or confers no added benefits. This was true in both clinical regimens and is consistent with findings from prior research [17]. These findings likely reflect some selection bias, however, since uterine massage is often provided in response to heavy post-partum bleeding. We suspect this may have occurred to some degree in our study, particularly among those for whom uterine massage was initiated either before or during placental delivery, rather than after as per current AMTSL guidelines [2]. Furthermore application of abdominal pressure may facilitate expulsion of blood into the measurement receptacle at a more rapid pace than would otherwise occur. If so, this may also explain at least some of the positive association between uterine massage and time dependent measures of post-partum blood loss, even if the intervention itself was not a source of increased bleeding.

Our analysis also provided an opportunity to examine the effect of route of oxytocin administration on post-partum blood loss. The results indicate that the longstanding assumption of equivalent effect may be incorrect, and that intravenous administration is superior to intramuscular administration. The evidence for this can be found in the large differences in hemorrhage risk among those who received oxytocin only. The fact that route of administration had no discernible effect on hemorrhage risk when oxytocin was combined with other active management interventions suggests that any underlying differences due to route of administration are mediated by the presence of controlled cord traction and possibly also by uterine massage.

While one of the key strengths of this analysis was the large number of participants in each AMTSL group with objectively measured blood loss post-partum, there were also several limitations. The studies were carried out in hospital facilities only, and thus may not be generalizable to other delivery settings. In addition, the provision of AMTSL components was not randomly assigned. Finally, there was some variation in procedures for administering AMTSL interventions, and these differences occurred both within- and between-sites. This variation is also a strength, however, since it reflects real differences in implementation of active management of the third stage of labor [24-27].

## Conclusions

Our findings call into question current consensus regarding the provision of AMTSL components and suggest that practice guidelines should account for setting-related differences such as the availability of oxytocin and its route of administration. The optimal combination of interventions will vary accordingly.

## Endnote

^a^See results listed at the bottom of Table 4 for additional tests for differences between AMTSL groups.

## Competing interests

All authors declare that we have no competing interests.

## Authors’ contributions

All authors contributed to data analysis, interpretation of results, and drafting of the manuscript. BW, JB and JD also contributed to conception, planning and carrying out of the original trials which provided the data for this analysis. All authors read and approved the final manuscript.

## Pre-publication history

The pre-publication history for this paper can be accessed here:

http://www.biomedcentral.com/1471-2393/13/46/prepub
